# Effect of Ca Content on Electrochemical Discharge and Corrosion Performance of Mg-6Al-1Sn Alloy Anodes for Mg-Air Batteries

**DOI:** 10.3390/ma18071562

**Published:** 2025-03-30

**Authors:** Xiaofeng Wan, Chenyuan Kang, Qiyuan Tian, Jingling Zhou, Shuangqing Qian, Chunhui Ma

**Affiliations:** 1 School of Mechanical Engineering, Nantong University, Nantong 226019, China; wan.xf@ntu.edu.cn (X.W.); m13773810945@163.com (C.K.); 2310110359@stmail.ntu.edu.cn (Q.T.); zhou.jl@163.com (J.Z.); sqqian@ntu.edu.cn (S.Q.); 2 School of Oceanography, Shanghai Jiao Tong University, Shanghai 200240, China

**Keywords:** Mg-6Al-1Sn, Ca, electrochemical corrosion, discharge performance, utilization efficiency

## Abstract

This study conducted a systematic investigation on how Ca content affected Mg-6Al-1Sn alloys as anodes for Mg-air batteries in terms of their microstructure, electrochemical corrosion behavior, and discharge performance. According to the investigation results, incorporating Ca induces the formation of blocky β-Mg_17_Al_12_ phases containing Ca and refines the grain structure. Compared to Mg alloys without Ca, the alloys with Ca exhibit significantly improved self-corrosion resistance because the preferential enrichment of Ca at the grain boundaries within β-Mg_17_Al_12_ phases reduces the potential difference between β-Mg_17_Al_12_ phases and Mg matrix. Consequently, galvanic corrosion is mitigated, together with the effective suppression of the self-corrosion reaction of Mg anodes. Additionally, Mg alloy shows a higher anode utilization efficiency with Ca content. The combined results indicate that Mg-6Al-1Sn-0.5Ca alloy exhibits superior self-corrosion resistance and discharge properties vs. other tested compositions. Furthermore, the Mg-air battery using Mg-6Al-1Sn-0.5Ca alloy as the anode demonstrates a heavier average discharge potential and a utilization efficiency of 71.12%, which is 7.56% higher than Mg-6Al-1Sn alloy.

## 1. Introduction

Mg-air batteries exhibit strong specific energy density, substantial specific capacity, environmental sustainability, safety, etc., proving their significant potential in the energy storage and conversion field [1,2,3,4,5]. Furthermore, Mg presents a negative standard electrode potential (−2.73 V), suggesting that Mg alloys exhibit high discharge activity and external output electrons to generate current [6,7,8]. In addition, due to the high reactivity of Mg, it is susceptible to oxidation and the formation of oxides on the surface, resulting in elevated anode polarization and inadequate anode utilization efficiency, which greatly reduces the intrinsic superior properties of Mg anode materials [9,10,11]. Additionally, the hydrogen evolution side reaction frequently occurs in the Mg alloy anode, resulting in heat generation, which leads to a reduction in specific electrode capacity and adversely impacts Mg-air batteries’ safety performance [12,13,14]. Despite the fact that Mg-air batteries theoretically possess a voltage of 3.1 V, the actual voltage observed during practical applications is significantly lower, which greatly restricts the widespread adoption of Mg-air batteries [15,16]. Alloying can potentially enhance the electrochemical property and discharge performance of Mg anodes through the modification of their microstructure and chemical composition [17,18,19].

The electrochemical property of the Mg-based anode is optimized by employing elements of Al, Li, Ca, Zn, Bi, Sn, and Mn [20]. Deng et al. revealed that Mg-0.1wt.%Ca alloys exhibit higher discharge potential and specific energy [21]. However, Mg-Ca binary alloys suffer from high self-corrosion, affecting performance. Xiong et al. found that Sn incorporation improves self-corrosion resistance by increasing the hydrogen evolution over-potential, triggering the formation of Mg_2_Sn, a phase with high thermal stability that enhances electrochemical performance by disrupting the passive film on the anode [22].

In pursuit of environmentally friendly alternatives, Mg-Al-Sn alloys exhibit superior mechanical properties and reduced toxicity compared to Pb-containing systems [23]. Yu et al. demonstrated that Mg-Al-Sn alloys exhibit higher discharge potential and energy density, improving battery performance [24]. Additionally, Zheng et al. confirmed through orthogonal design studies that increasing the Mn content in Mg-Al-Sn alloys resulted in a more uniform distribution of corrosion products, reduced self-corrosion, and significant grain refinement [25]. According to Miao et al., in Mg-2.0wt.%Al-0.8wt.%Sn alloys, an increase in Ca content enhances strength and thermal stability due to the formation of CaMgSn phases [26].

Despite the impact of Ca on Mg-Al-Sn-based alloys in terms of discharge behavior, a systematic investigation of this relationship has yet to be conducted. The study has the objective of examining how Ca concentrations affect the alloys’ electrochemical properties and the correlation between the microstructure evolution and corrosion properties.

## 2. Materials and Methods

### 2.1. Materials Preparation

Mg-6Al-1Sn-xCa (x = 0.1, 0.3, 0.5wt.%) anodes were prepared by melting pure Mg (99.9wt.%), pure Al (99.9wt.%), pure Sn (99.9wt.%), and Mg-30%Ca, Al-10%Mn intermediate alloys under CO_2_ (bal) and SF_6_ (1%). Then, a cylindrical sample was made by pouring the molten metal into a water-cooled mold. After cooling, a 10 mm × 10 mm × 10 mm sample made from the center of the cylindrical sample was obtained via wire-cutting.

### 2.2. Microstructure Analysis

Analysis of the Mg-6Al-1Sn-xCa alloy composition relied on inductively coupled plasma-atomic emission spectroscopy (ICP-AES) (Table 1) [27]. The samples had to be cold-mounted, ground, and polished until there were no scratches before observation. 

GeminiSEM300 scanning electron microscope (Carl Zeiss AG, Oberkochen, Germany) with energy dispersive spectroscopy (EDS) and SGO-130VRX optical microscopy (Shenzhen Shenshi Optical Valley Optical Instrument Co., Ltd., Shenzhen, China) characterized the surface morphology and structure and determined the sample composition. D-MAX2500/PC X-ray diffraction (Tokyo, Japan) assisted in analyzing the prepared alloys’ physical phase composition.

### 2.3. Electrochemical Measurements and Corrosion Test

A three-electrode system encompassing a working electrode, an auxiliary electrode (platinum electrode), and a reference electrode (calomel electrode) served for the electrochemical measurements. An open-circuit potential (OCP) test was required to acquire a stable OCP prior to the electrochemical test, i.e., the sample was immersed in the solution for 30 min. The polarization curve was obtained by scanning the anodic potential in intervals of 500 mV from the radius of the open-circuit potential at a rate of 0.1 mV/s. Electrochemical impedance spectroscopy (EIS) measurements were performed under OCP in a frequency range of 0.01 Hz to 100 kHz. Then, Nyquist plots were synthesized for analysis by Zview 3.1 software.

The tested area specific to each working electrode was reserved as 1 cm^2^ and polished to 2000#. All electrochemical experiments were determined by the CHI660C electrochemistry workstation in 3.5wt.% NaCl solution at 27 °C and repeated 5 times to ensure experimental accuracy.

### 2.4. Immersion Experiments

The self-corrosion properties were tested by immersion experiments, i.e., immersing the samples in the 3.5wt.% NaCl electrolyte for 36 h at 27 °C. The completion of corrosion was followed by the cleaning of the surface corrosion products using chromic acid. The equations below were used to interpret the self-corrosive reaction [28]:

(1) $$Mg\to {Mg}^{2+}+{2e}^{-}$$

(2) $$2H_{2}O+{2e}^{-}\to H_{2}+{2OH}^{-}$$

(3) $${Mg}^{2+}+{2OH}^{-}\to {Mg(OH)}_{2}$$

The erosive action of ${Cl}^{-}$ destroys the passivation film ($Mg(OH{)}_{2}$) and results in local corrosion, the reaction of which is as follows:

(4) $${Mg(OH)}_{2}+{2Cl}^{-}\to {MgCl}_{2}+{2OH}^{-}$$

The addition of Ca inhibits the hydrogen precipitation reaction as follows:

(5) $${Ca}^{2+}+{2OH}^{-}\to {Ca(OH)}_{2}$$

The equation below explains the calculation of the self-corrosion rate [29]:

(6) $$V=\frac{M_{0}-M_{1}}{s\times t}$$

where V represents the self-corrosion rate (g m^−2^h^−1^), $M_{0}$ and $M_{1}$ are the initial and post-corrosion weight of alloys (g), respectively, s is the sample surface area (cm^2^), and t is the corrosion time (s).

The hydrogen evolution rate is calculated by collecting the volume of hydrogen precipitated from the Mg alloy during self-corrosion, as shown in the equation below [30]. The volume of hydrogen precipitated from the solution was collected every two hours using the drainage method, as shown by the change in the liquid level of the glass tube.

(7) $$V_{H}=\frac{∆V}{s\times t}$$

where $V_{H}$ and ∆V refer to the hydrogen evolution rate (mL cm^−2^ h^−1^) and volume (mL), respectively. s and t represent the same factors as the previous equation.

### 2.5. Discharge Performance

The anode discharge performance was tested using a three-electrode system consisting of a working electrode, an auxiliary electrode (platinum electrode), and a reference electrode (calomel electrode). The constant current discharge curves pertaining to the samples discharged in the 3.5wt.% NaCl electrolyte for 1000 s were measured using the constant current method with applied anode currents of 5, 10, 30, and 60 mA^−2^. After discharge, SEM assisted in visualizing the surface morphology while ensuring that the discharge products were removed. The equations below interpret the mass loss method for calculating the anode utilization efficiency of the magnesium anode [31,32]:

(8) $$\eta =\frac{M_{t}}{M_{a}}\times 100\%$$

(9) $$M_{t}=\frac{I\times t}{F\times \sum \left(\frac{X_{i}\times n_{i}}{m_{i}}\right)}$$

where η denotes the anode utilization efficiency (%), $M_{t}$ and $M_{a}$ are the theoretical and actual mass loss (g), I represents the operating current (A cm^−2^), t represents the discharge time (s), F is Faraday’s constant (96,485 C·mol^−1^), and $X_{i}$, $n_{i}$, and $m_{i}$ denote the element mass fraction, ionic valence, and molar mass (g·mol^−1^). All measurements were repeated ≥3 times to ensure the accuracy of the results.

## 3. Results

### 3.1. Microstructure

**Figure 1:**
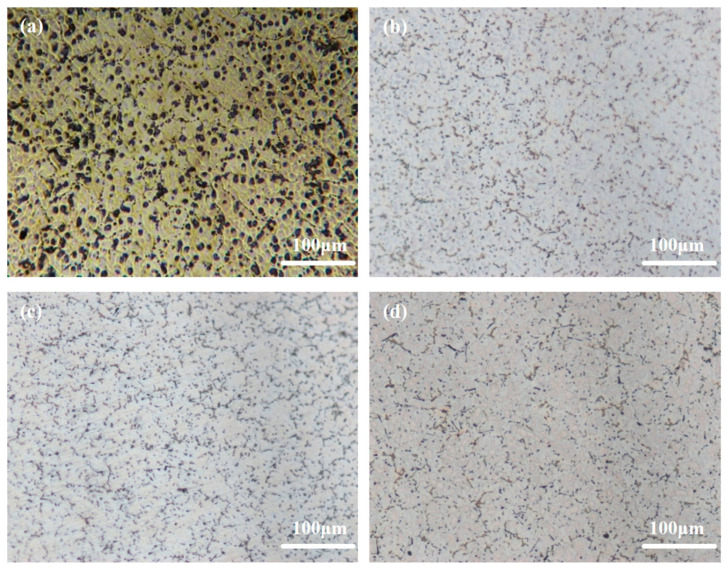
Optical microstructures of Mg-6Al-1Sn-xCa alloys: (**a**) AT61; (**b**) ATCa6101; (**c**) ATCa6103; (**d**) ATCa6105.

Figure 1 illustrates the optical images pertaining to Mg-6Al-1Sn-xCa alloys with varying Ca content. The matrix structure of the alloy is significantly refined with the addition of 0.1wt.% Ca, causing a smaller grain size of the granular phase (Figure 1b). As Ca content is further increased, the granular phase continues to be refined, exhibiting a uniform and discrete distribution (Figure 1c,d). A semi-continuous net-like structure is distinctly observable in Figure 1d at a Ca content of 0.5wt.%. These results align with the theoretical understanding that Ca acts as an effective inhibitor of grain growth in Mg alloys, effectively impeding the growth of grains, with its inhibitory effect being second only to Zr [33].

**Figure 2:**
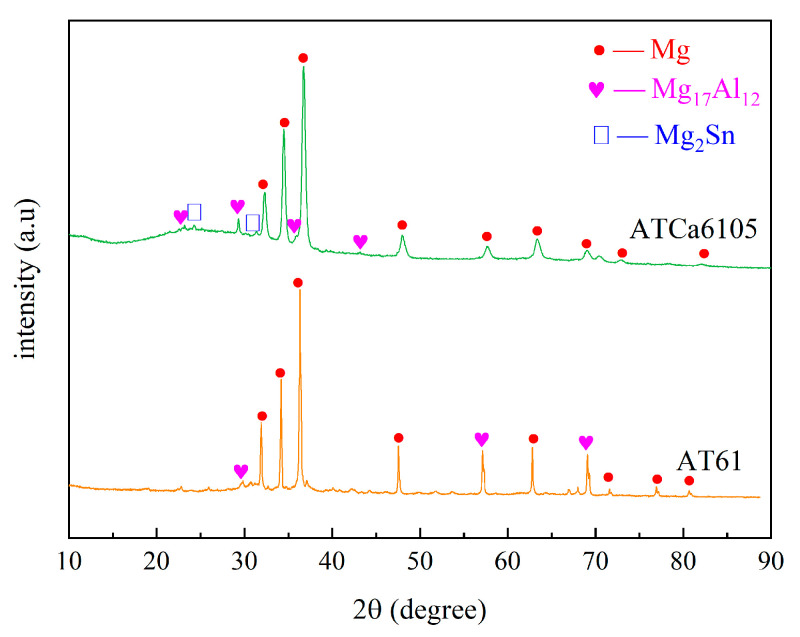
XRD spectrum of AT61 and ATCa6105.

**Figure 3:**
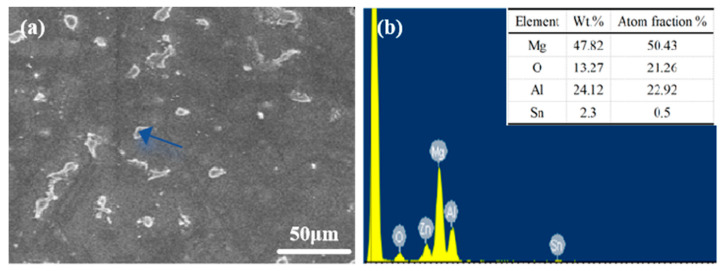
(**a**) SEM images of AT61 alloy; (**b**) EDS spectrum of the area indicated by the arrow in figure (**a**).

According to Figure 2 and Figure 3, the Mg matrix is predominantly composed of substantial quantities of Al and trace amounts of Sn. The as-cast microstructure of AT61 alloy primarily comprises an α-Mg solid solution and a granular β-Mg_17_Al_12_ phase enriched with Sn.

**Figure 4:**
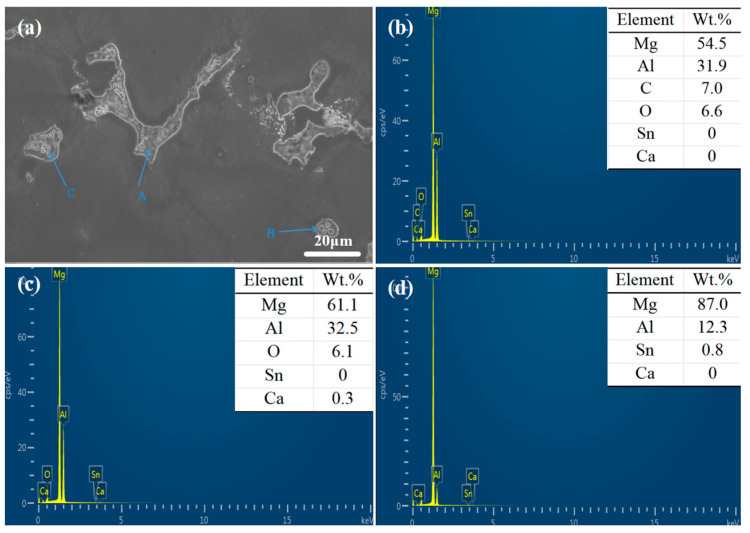
SEM images and EDS spectrum of ATCa6105 alloy: (**a**) microstructure; (**b**) Position A; (**c**) Position B; (**d**) Position C.

The XRD spectrum and EDS analysis of each precipitated phase of the ATCa6105 alloy (Figure 2 and Figure 4) indicate that the incorporation of Ca leads to the development of skeletal and blocky structures. These structures can be identified as β-Mg_17_Al_12_ phases that contain both Sn and Ca. Additionally, ATCa6105 alloys exhibit granular phases with compositions comprising Mg, Al, and Ca, as observed in composition B. These granular phases can be interpreted as β-Mg_17_Al_12_ intermetallic compounds modified by Sn, which aligns with the phases in Figure 3 and Figure 4c. Additionally, the composition at point C is suggested to correspond to the Mg_2_Sn phase containing Ca, characterized by a substantial amount of Mg, a moderate presence of Al, and trace amounts of Sn.

### 3.2. Electrochemical Performance

#### 3.2.1. Potentiodynamic Polarization Curves

**Figure 5:**
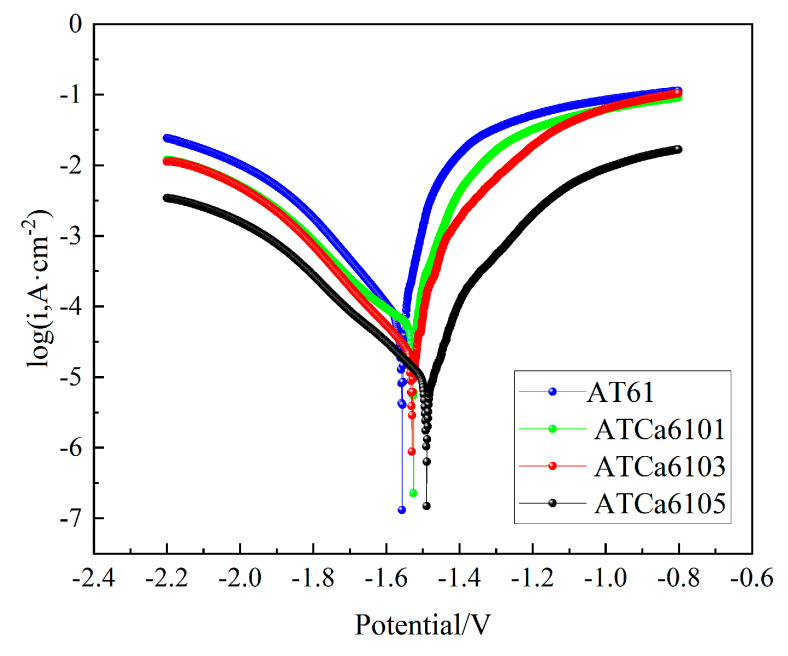
Potentiodynamic polarization curves of investigated alloys in 3.5wt.% NaCl solution at 27 °C.

Figure 5 provides the potentiodynamic polarization curves of all investigated alloys. The alloy incorporated with Ca has the corrosion potential shifted in a positive direction. The Tafel intersection method was employed to determine the corrosion potential ($E_{corr})$ and corrosion current density $\left(j_{corr}\right)$ (Table 2). $E_{corr}$ of AT61 alloy is the most negative (−1.557 V vs. standard calomel electrode (SCE)) among the investigated alloys, whereas ATCa6105 alloy shows the most positive $E_{corr}$ (−1.492 V vs. SCE). However, $E_{corr}$ for alloys with 0.1wt.% and 0.3wt.% Ca remains relatively close. In comparison to $j_{corr}$ of AT61 (1.126 × 10^−4^ A·cm^−2^), ATCa6105 alloy (9.43 × 10^−6^ A·cm^−2^) is reduced by two orders of magnitude, the anodic Tafel slope of which is found to be larger than that of other alloys (12.74), consistent with the results of the hydrogen precipitation experiments, which showed that the volume of precipitated hydrogen and the rate of precipitation for the self-corrosion precipitation at the anode were lower than those of the other alloy anodes. Hence, the alloy with 0.5wt.% Ca added shows the optimal corrosion, which is because a Ca-containing second phase is formed. The incorporation of Ca into the alloy tends to form a second phase concentrated at the grain boundaries. Alloys containing β-Mg_17_Al_12_ phases, which form upon the addition of Ca, have a negative second-phase potential, thereby reducing the potential difference between the β-Mg_17_Al_12_ phase and the Mg matrix. This reduction enhances the mitigation of microgalvanic corrosion. Furthermore, the self-corrosion reaction of the Mg anode can be suppressed by preventing the formation of an oxide film on the surface.

#### 3.2.2. EIS

**Figure 6:**
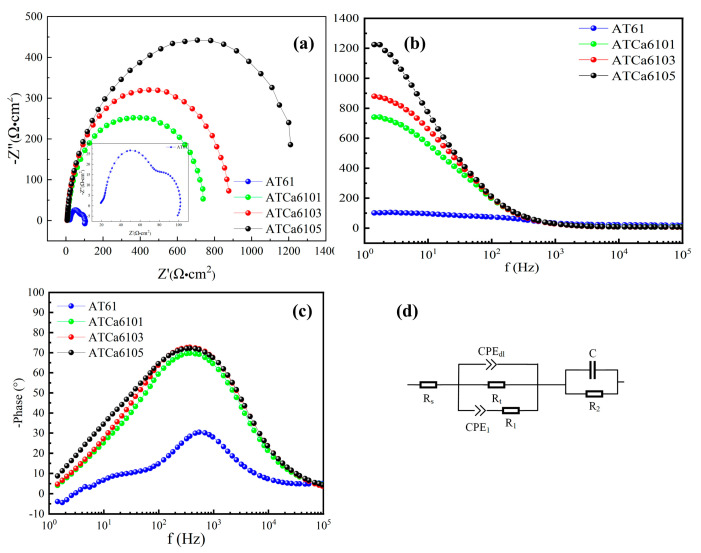
EIS analysis of Mg alloys in 3.5wt.% NaCl solution: (**a**) Nyquist plots; (**b**) Bode magnitude plots; (**c**) phase angles; (**d**) equivalent circuits.

Figure 6 presents EIS results for samples with varying Ca contents at OCPs. For the Nyquist plot of the anodes in Figure 6a, elevated Ca content results in a longer impedance arc radius, suggesting that Mg alloys exhibit dramatically reduced discharge activity after having Ca added. The inductive effect obtained in AT61 at low frequencies is due to the adsorption and desorption of corrosion products and Cl-induced local corrosion. Furthermore, Nyquist plots for the examined anodes predominantly present a capacitive reactance arc in the high-frequency region, which relates to the charge transfer, where the difficulty of charge transfer is positively correlated with the Bode phase angle (Figure 6c). There are no detectable inductive arcs in the low-frequency and mid-frequency regions because of the minimal shedding of corrosion product films, indicating an overall enhancement in corrosion resistance with the incorporation of Ca. The trends in corrosion performance, as derived from the Bode magnitude plots (Figure 6b), align with those observed in Nyquist plots. A larger Bode phase angle corresponds to increased difficulty in charge transfer, a greater diameter of the capacitive reactance arc, and consequently, improved corrosion resistance. Therefore, ATCa6105 alloy, which exhibits the largest Bode phase angle, demonstrates superior corrosion resistance.

Figure 6d illustrates the equivalent circuit utilized to analyze EIS data. Among the various Mg alloy samples examined, the charge transfer resistance ($R_{t}$) of ATCa6105 alloy, as determined from EIS-fitting results (Table 3), is the highest, measuring approximately 540.61 Ω·cm^2^. This elevated $R_{t}$ correlates with a reduced corrosion rate and enhanced discharge performance. Therefore, Mg alloys incorporated into Ca exhibit obviously stronger corrosion resistance.

### 3.3. Self-Corrosion Rate

Self-corrosion in Mg-6Al-1Sn alloy anodes during charge and discharge, attributed to the reduction–oxidation process, is a significant factor that diminishes the utilization efficiency of Mg batteries. Therefore, it is essential to investigate the self-corrosion rate.

**Figure 7:**
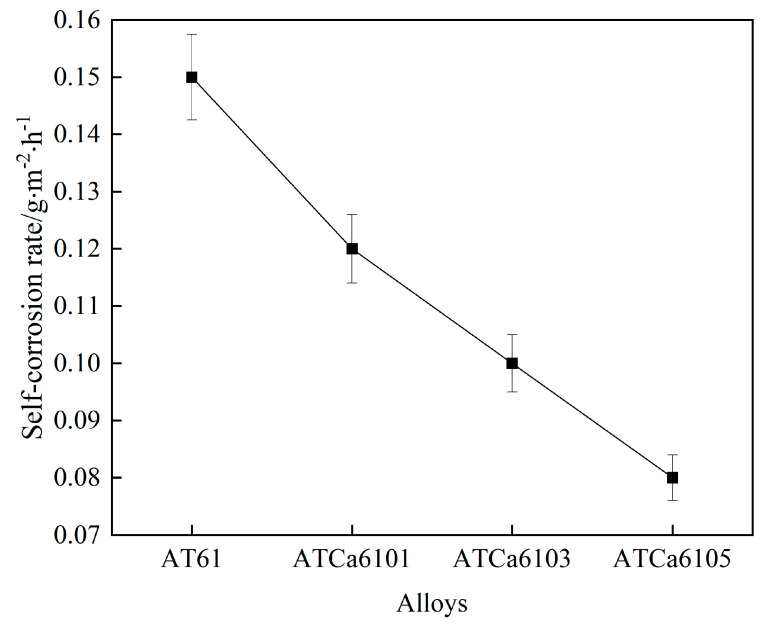
Self-corrosion rates of all investigated alloys.

We measured the actual surface area of all examined alloys and determined the self-corrosion regarding Mg anodes after 36 h of immersion in a 3.5wt.% NaCl solution, based on the mass loss over the corrosion duration. Figure 7 and Table 4 illustrate the results. The incorporation of Ca content reduces the average corrosion rate of the samples, suggesting that Ca plays a “corrosion inhibition” role within the electrode. Mg alloys present decreasing self-corrosion rates as Ca content increases, with the alloy exhibiting the lowest self-corrosion rate and enhanced corrosion resistance with the addition of 0.5wt.% Ca.

### 3.4. Hydrogen Evolution Measurement

**Figure 8:**
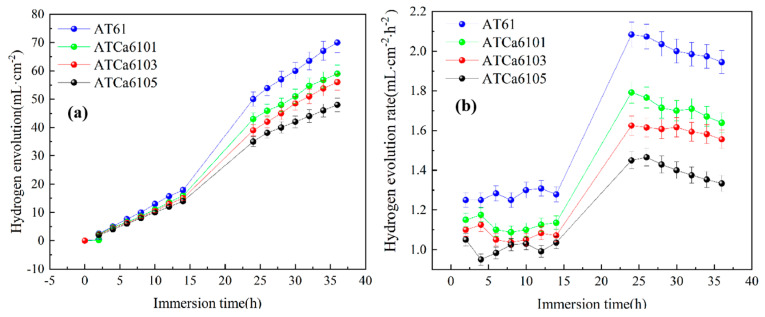
Hydrogen evolution behavior of Mg alloys immersed in 3.5wt.% NaCl at 27 °C: (**a**) hydrogen evolution volume; (**b**) hydrogen evolution rate.

The hydrogen evolution reaction resulting from the Mg alloy self-corrosion is an important metric for the corrosion performance assessment of micro-alloyed anodes. The variation curves depicting both the volume and rate of hydrogen evolution during this process are illustrated in Figure 8. In Figure 8a, the hydrogen evolution volumes increase with soaking time. Notably, the hydrogen evolution volume and rate associated with the self-corrosion of the ATCa6105 anode are lower than those observed in other alloy anodes. Hence, the second phase that Ca exerts can markedly inhibit the hydrogen evolution reaction of Mg anodes. The refinement of α grains occurs due to the formation of Mg_2_Sn in the presence of added Ca, which contributes to a reduction in the positive segregation of Al from the α intragranular regions to the grain boundaries. This grain refinement not only facilitates the maintenance of high Al content but also diminishes the inhomogeneity at the grain boundaries, hence intensifying the alloy’s corrosion resistance. Furthermore, the alloy containing 0.5wt.% Ca exhibits a relatively low hydrogen evolution rate, corroborating the results from polarization curve analysis and indicating that ATCa6105 alloy exhibits commendable corrosion resistance.

### 3.5. Discharge Performance

#### 3.5.1. Surface Micro-Morphology Analysis After Discharge

**Figure 9:**
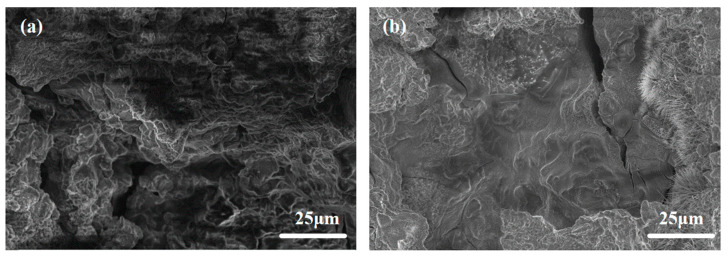
SEM morphology of Mg alloys after 4 h of discharge in 3.5wt.% NaCl at 10 mA·cm^−2^: (**a**) ATCa6101; (**b**) ATCa6105.

Figure 9 shows the SEM morphologies of all investigated alloys after 4 h of discharge in 3.5wt.% NaCl at a current density of 10 mA·cm^−2^. The surface of ATCa6101 exhibits significant corrosion, characterized by pronounced irregularities, including large holes and cracks, which may adversely impact the anode utilization efficiency regarding Mg alloys (Figure 9a). Comparatively, the surface corrosion products of ATCa6105 are comparatively minimal, with no significant intergranular corrosion observed post-discharge (Figure 9b). Furthermore, the central region of the Mg matrix appears relatively uniform, exhibiting less corrosion and only a few shallow crack-like corrosion features. On these accounts, incorporating 0.5wt.% Ca causes alloy anodes to be more uniformly dissolved and effectively mitigates the corrosion of the Mg matrix by the electrolyte during prolonged low-current discharge conditions. In addition, the Ca(OH)_2_ layer formed on the anode surface resulting from the CaSn phase dissolution inhibits the corrosion rate.

#### 3.5.2. Discharge Characteristics

**Figure 10:**
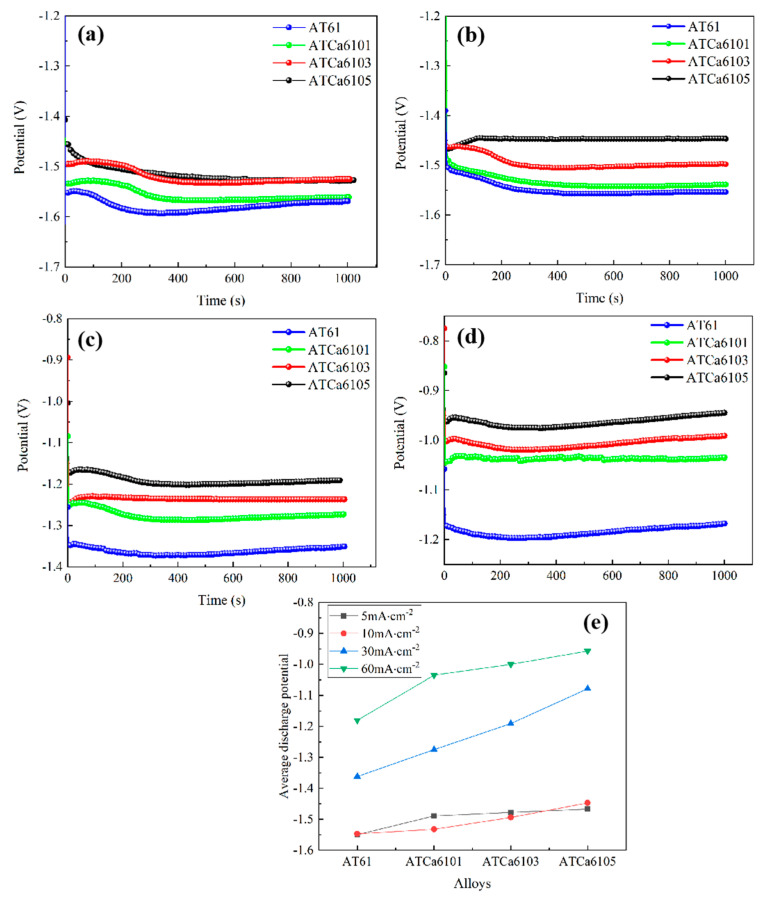
Current density-dependent discharge curves of Mg alloy electrodes: (**a**) 5 mA/cm^2^; (**b**) 10 mA/cm^2^; (**c**) 30 mA/cm^2^; (**d**) 60 mA/cm^2^; (**e**) average discharge potential.

Figure 10 shows the discharge curves of all investigated alloy anodes at different anodic current densities. It can be seen that the discharge potential generally shifts in a positive direction as the current density rises, and the discharge curves exhibit stability. According to Zheng et al. [25], a more negative average discharge potential during constant current discharge testing indicates enhanced discharge activity. Furthermore, a smaller fluctuation in the discharge curve correlates with the improved stability of the Mg anode battery. The stable discharge curve observed for Mg alloy suggests that the formation and detachment of surface corrosion products of the electrode in the discharge process have reached dynamic equilibrium. In Figure 10e, Mg alloys exhibit a more positive discharge potential as Ca content increases. Notably, ATCa6105 alloy demonstrates the most positive discharge potential across varying current densities, recording −1.550 V at 5 mA·cm^−2^ compared to −1.180 V at 60 mA·cm^−2^. Additionally, its discharge curve is the most stable, indicating that the incorporation of 0.5wt.% Ca effectively reduces the alloy discharge activity and makes the discharge process more stable.

**Figure 11:**
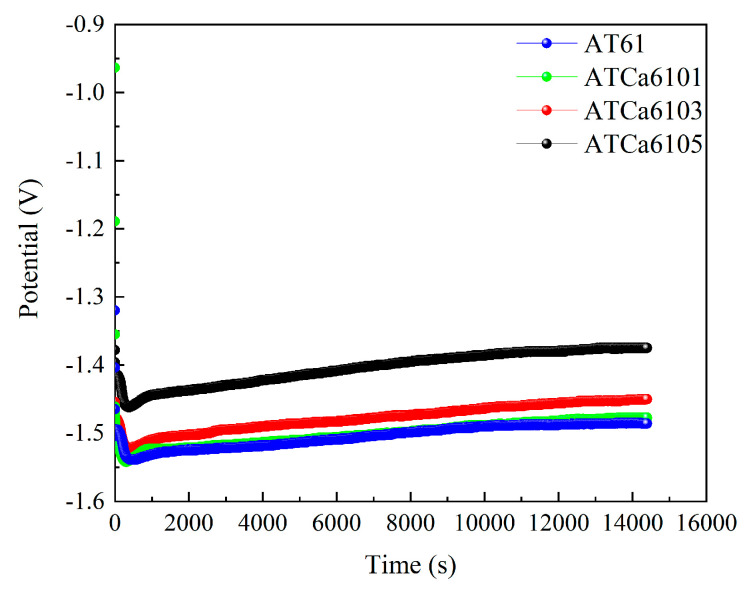
Discharge curves of all investigated alloy electrodes at 10 mA cm^−2^ for 4 h.

**Figure 12:**
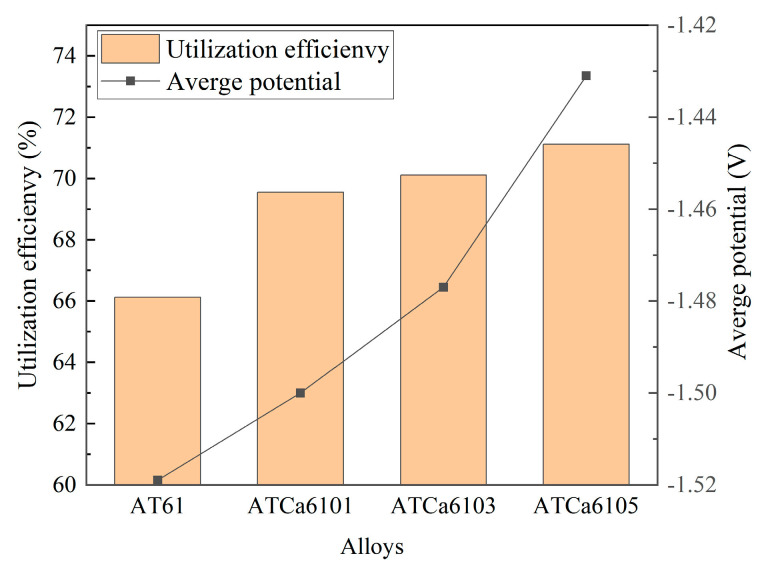
Average discharge potential and utilization efficiency of Mg alloy electrode at 10 mA cm^−2^ for 4 h.

Figure 11 shows the discharge curves of all investigated alloy anodes, which were discharged for 4 h at a current density of 10 mA·cm^−2^ in a 3.5wt.% NaCl solution. The average discharge potential and utilization efficiency are illustrated in Figure 12. ATCa6105 alloy possesses the most positive average discharge potential (−1.431 V) and a high discharge efficiency of 71.12% because the Ca-containing phase formed on the electrode surface effectively facilitates the breaking down of the Mg(OH)_2_ passivation film during the discharge process. Consequently, this results in more rapid dissolution of the alloy compared to other Mg anodes with various Ca contents. Therefore, Mg alloy with 0.5wt.% Ca added is more conducive to promoting utilization efficiencies.

## 4. Conclusions

Mg-6Al-1Sn alloys with 0.1wt.%, 0.3wt.%, and 0.5wt.% Ca in a 3.5wt.% NaCl solution were subjected to electrochemical measurements together with discharge tests to examine their microstructure, electrochemical corrosion, and discharge performance. The results reveal that the introduction of Ca into Mg-Al-Sn alloy can refine the grains and promote the formation and homogeneous distribution of blocky β-Mg_17_Al_12_ phases containing Sn and Ca. The alloys present significantly enhanced corrosion resistance since Ca alloying reduces the hydrogen evolution rate. Additionally, the alloys exhibit more positive average discharge potentials and superior discharge stability as Ca content increases. Among the alloys tested, the 0.5wt.% Ca-added alloy demonstrates the best discharge performance, with an anode utilization efficiency reaching 71.12% at 10 mA·cm^−2^. Such exceptional performance results from the ability of the Ca alloy to promote the uniform dissolving of the anodes, ensuring consistently dense corrosion pores. This characteristic enhances the peeling of discharge products, thereby improving the anode utilization efficiency.

## Figures and Tables

**Table 1:** Mg-6Al-1Sn-xCa anode chemical composition.

Anode Designation (Code)	Chemical Composition (wt.%)				
	Al	Sn	Ca	Mn	Mg
Mg-6Al-1Sn (AT61)	5.87	1.12	-	0.27	bal
Mg-6Al-1Sn-0.1Ca (ATCa6101)	5.78	0.98	0.12	0.29	bal
Mg-6Al-1Sn-0.3Ca (ATCa6103)	6.12	0.93	0.28	0.28	bal
Mg-6Al-1Sn-0.5Ca (ATCa6105)	5.89	0.95	0.53	0.26	bal

**Table 2:** $E_{corr}$ and $j_{corr}$ of investigated alloy electrodes.

Alloy	$E_{corr}$ (V vs. SCE)/V	$j_{corr}$/(A/cm^2^)
AT61	−1.557	1.126 × 10^−4^
ATCa6101	−1.523	5.598 × 10^−5^
ATCa6103	−1.525	2.969 × 10^−5^
ATCa6105	−1.492	9.43 × 10^−6^

**Table 3:** Electrochemical parameters obtained by fitting analysis of different alloys.

Alloy	AT61	ATCa6101	ATCa6103	ATCa6105
R_S_/Ω·cm^2^	0.01	2.837	3.093	4.815
CPE_dl_/Ω^−1^·cm^−2^·s^n^	1.054 × 10^−4^	1.013 × 10^−5^	1.413 × 10^−5^	1.473 × 10^−6^
R_t_/Ω·cm^2^	52.1	404.3	473.7	540.61
CPE_1_/Ω^−1^·cm^−2^·s^n^	3.334 × 10^−4^	1.093 × 10^−5^	2.297 × 10^−6^	1.679 × 10^−6^
R_1_/Ω·cm^2^	8.133	6.826	10.42	6.681
C/μF	1.004 × 10^−5^	6.111 × 10^−5^	5.147 × 10^−5^	4.31 × 10^−5^
R_2_/Ω·cm^2^	18.1	4.361	6.594	2.067

**Table 4:** Measured surface area of all investigated alloys.

Component	Initial Mass/mg	Final Mass/mg	Actual Measurement/mm^2^
AT61	1335.3	1339.8	10.75 × 10.70
ATCa6101	1331.4	1329.8	9.65 × 9.67
ATCa6103	1441.5	1437.9	9.48 × 9.60
ATCa6105	1394.9	1388.4	9.60 × 9.78

## Data Availability

The original contributions presented in the study are included in the article. Further inquiries can be directed to the corresponding author.
